# Mesopancreatic Stromal Clearance Defines Curative Resection of Pancreatic Head Cancer and Can Be Predicted Preoperatively by Radiologic Parameters: A Retrospective Study

**DOI:** 10.1097/MD.0000000000002529

**Published:** 2016-01-22

**Authors:** Ulrich F. Wellner, Tobias Krauss, Agnes Csanadi, Hryhoriy Lapshyn, Louisa Bolm, Sylvia Timme, Birte Kulemann, Jens Hoeppner, Simon Kuesters, Gabriel Seifert, Dirk Bausch, Oliver Schilling, Yogesh K. Vashist, Thomas Bruckner, Mathias Langer, Frank Makowiec, Ulrich T. Hopt, Martin Werner, Tobias Keck, Peter Bronsert

**Affiliations:** From the Clinic for Surgery, UKSH Campus Lübeck, Lübeck (UFW, HL, LB, DB, TK); Clinic for Radiology (TK, ML); Institute of Pathology (AC, ST, MW, PB); Clinic for General and Visceral Surgery, University Medical Center Freiburg (BK, JH, SK, GS, FM, UTH); Institute for Molecular Medicine and Cell Research, University of Freiburg, Freiburg (OS); Department of Surgery, University Hospital Hamburg-Eppendorf (UKE), Hamburg (YKV); Institute of Medical Biometry and Informatics (IMBI), University of Heidelberg, Heidelberg (TB); Comprehensive Cancer Center Freiburg, Freiburg (ML, FM, UTH, MW, PB); and German Cancer Consortium (DKTK) and German Cancer Research Center (DKFZ), Heidelberg, Germany (OS, MW, PB).

## Abstract

Pancreatic ductal adenocarcinoma (PDAC) is characterized by a strong fibrotic stromal reaction and diffuse growth pattern. Peritumoral fibrosis is often evident during surgery but only distinguishable from tumor by microscopic examination. The aim of this study was to investigate the role of clearance of fibrotic stromal reaction at the mesopancreatic resection margin as a criterion for radical resection and preoperative assessment of resectability.

Mesopancreatic stromal clearance status (S-status) was defined as the presence or absence (S+/S0) of fibrotic stromal reaction at the mesopancreatic resection margin. Detailed retrospective clinicopathologic re-evaluation of margin status and preoperative cross-sectional imaging was performed in a cohort of 91 patients operated for pancreatic head PDAC from 2001 to 2011.

Conventional margin positive resection (R+, tumor cells directly at the margin) was found in 36%. However, S-status further divided the margin negative (R0) group into patients with median survival of 14 months versus 31 months (S+ versus S0, *P* = 0.005). Overall rate of S+ was 53%. S-status and lymph node ratio constituted the only independent predictors of survival. Stranding of the superior mesenteric artery fat sheath was the only independent radiologic predictor of S+ resection, and achieved a 71% correct prediction of S-status.

Mesopancreatic stromal clearance is a major determinant of curative resection in PDAC, and preoperative prediction by cross-sectional imaging is possible, setting the basis for a new definition of borderline resectability.

## Abstract 2

Supplemental Digital Content is available in the text

## INTRODUCTION

Only about 20% of patients with pancreatic ductal adenocarcinoma (PDAC) present with localized disease amenable to surgical resection.^1^ PDAC is characterized by fast local progression and early distant metastasis resulting in one of the worst survival rates among all human cancers.^2,3^ Even successful surgical resection yields 5-year survival rates of only around 20%.^4,5^ Moreover, patients continue to succumb to the disease even after prolonged survival (>5 years).^6^ Virtually all patients experience local and/or metastatic recurrence.^7–9^

The mere anatomical location of pancreatic head cancer makes conventional radical resection of the local disease, obtaining wide security margins, nearly impossible. Only a narrow space posterior to the pancreatic head and neck separates the pancreas from the adjacent major arterial vessels. This space has been coined “mesopancreas,”^10,11^ although no clear connective tissue sheaths exist to fully justify the term “meso.”^11–13^ The mesopancreas contains variable amounts of fatty tissue, parts of the autonomous visceral nervous system, lymphatic vessels, and locoregional lymph nodes. It has been shown^10–13^ that most margin positive pancreatoduodenectomies are caused by margin positivity in the mesopancreatic area.^14–17^

While en bloc resection of the superior mesenteric or portal vein (PVR) for tumor adhesion has become standard in many centers,^18–21^ the superior mesenteric artery (SMA), hepatic artery (HA), inferior caval vein (ICV), and aorta are usually not considered resectable.^18,22^ Of note, preoperative prediction of resectability by radiologic criteria is sensitive and specific for portal venous, but only to a lesser degree for arterial involvement, where surgical exploration is usually needed in equivocal cases.^23,24^

During resection, the surgeon is often confronted with extensive peritumoral fibrotic stromal reaction in the mesopancreatic region.^17^ This results in adhesion to the aforementioned blood vessels and intraoperatively suggests borderline resectability of this tumor. In this situation, sharp dissection right through the fibrotic stroma is often necessary to mobilize the tumor and pancreatic head. Histopathologically, PDAC is characterized by an abundant fibrotic stromal reaction.^1,25,26^ The role of this peritumoral stroma is currently debated: experimental evidence points toward a supporting role of this stroma in the process of tumor invasion, metastasis, and treatment resistance, however contradictory results have also been published.^27,28^

Within this context, we strove to investigate the clinical impact of surgical mesopancreatic stromal clearance during resection of pancreatic head cancer. Furthermore, the aim was to predict the achievement of stromal clearance preoperatively by radiologic parameters.

## METHODS

### Patients and Tissue

Patients operated for pancreatic head PDAC from 2001 to 2011 at the Clinic for General and Visceral Surgery, University Medical Center Freiburg, with histopathological workup at the Institute of Pathology, University Medical Center Freiburg were identified. Permission was obtained from the institutional ethics committee of the University of Freiburg (ref 13/11). All histological samples and corresponding pathological reports were reevaluated independently by 3 experienced pathologists for correctness of diagnosis and resection margin status. Two experienced surgeons reviewed operation reports, clinical and follow-up data. Cases with perioperative death or insufficient material for detailed reevaluation were excluded.

### Standard Specimen Workup

A standardized workup for gross sectioning was performed for pancreatoduodenectomy. The biliary, oral and aboral enteric, pancreatic parenchymal, and mesopancreatic resection margins were marked by the surgeon. Extra tissue samples were evaluated on clinical demand. All specimens were transferred to the Institute of Pathology for frozen sectioning and examined by experienced pathologists. Macroscopic tumor masses were measured, tumor localization, infiltration of surrounding structures, and distance to resection margins were documented. The mesopancreatic margin was inked before gross sectioning for histologic orientation. The standard gross sectioning protocol comprised samples of the oral and aboral enteral, biliary, pancreatic circumferential, and parenchymal resection margins, as well as tumor samples in relation to the closest mesopancreatic margin, distal bile duct, main pancreatic duct, Ampulla of Vater, duodenum, and at least 12 locoregional lymph nodes. Tissue samples from the mesopancreatic margin and tumor samples were sliced orthogonal to the resection plane, and parallel to the resection plane from the parenchymal and proximal biliary margin. Resection margins of the splenic artery and vein and 1 sample of the spleen were taken in case of a total pancreatosplenectomy. In case of en bloc superior mesenteric or portal vein resection, tissue specimens of the vessel margins and tumor in relation to the vessel were embedded.

Tissue specimens were formalin fixed and paraffin embedded (FFPE) and 3-μm FFPE tissue slices were haematoxylin and eosin (H&E) stained according to a routine protocol. In case of detection of suspicious cells at the resection margins, immunohistochemistry for Pan-Cytokeratin was performed. Lymph nodes were evaluated separately. The histopathological report included tumor size, grade and WHO type,^29^ UICC staging (pTNM),^30^ and microscopic status of all evaluated resection margins. Furthermore, microscopic lymphangiosis (L), hemangiosis (V), and perineural invasion (Pn) were documented.

### Conventional Histopathologic Revalidation

**FIGURE 1:**
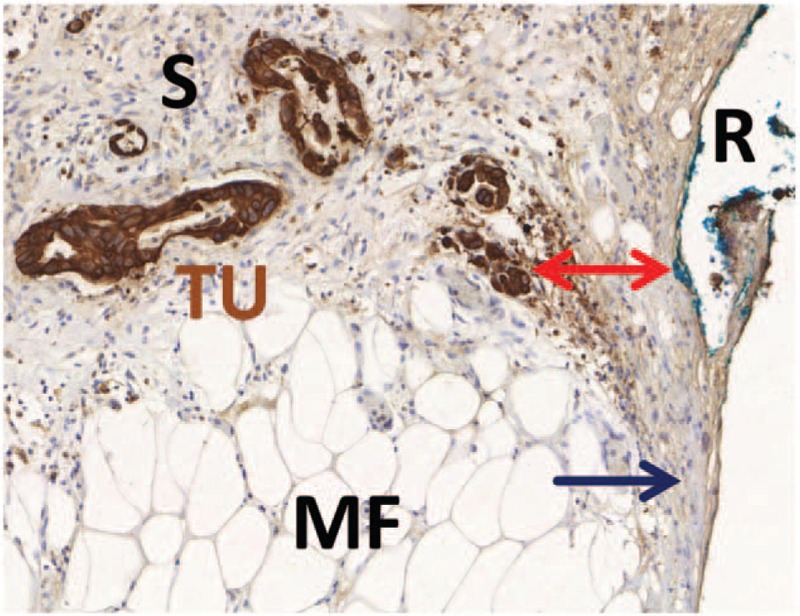
Conventional and histopathological margin status assessment. Example of a tissue slide from the mesopancreatic margin with brown immunohistochemical staining for Pan-Cytokeratin for better visualization of tumor cells. The tumor cells (TU) are surrounded by a dense fibrotic stroma (S) and invade the mesopancreatic fatty tissue (MF). The closest distance to the inked resection margin (R) is marked by a red arrow. Although no tumor cells are found directly at the resection margin, there is broad contact of the fibrotic stroma to the resection margin. Margin status in this case is negative by conventional R-status (R0, zero tumor cell distance rule), but positive by circumferential margin concept (CRM+, 1-mm tumor cell distance rule) and positive by stromal clearance concept (S+, zero stroma distance rule). For details see manuscript text.

All macroscopic and microscopic reports were reviewed independently by 3 experienced pathologists. Reexamination of the H&E stained tissue slides from the tumor and resection margins was performed at 200 and 400-fold magnification. Each resection margin was considered separately. Conventional resection margin status (R-status) was considered positive (R+) when tumor cells were found directly at any margin (zero tumor cell distance rule, see Figure 1).

### Reassessment of Mesopancreatic Resection Margin Status

H&E stained tissue slides of the mesopancreatic margins including samples from portal vein resections were reevaluated in blinded fashion by 2 experienced pathologists and 1 trained surgeon. The mesopancreatic stromal clearance status (S-status) was considered positive (S+) when fibrotic tissue containing fibroblasts and variable amounts of inflammatory infiltrate was identified directly at the inked margin (zero stroma distance rule, Figure 1). Inter-rater agreement for S-status was measured by assessment from 2 independent observers. Margin status according to the circumferential margin (CRM) concept, as originally derived from rectal cancer and adopted to pancreatic cancer,^17^ was considered positive (CRM+) when tumor cells were found within 1 mm of the inked margin (1-mm tumor cell distance rule, Figure 1).

### Assessment of Cross-Sectional Imaging for Prediction of Margin Status

**FIGURE 2:**
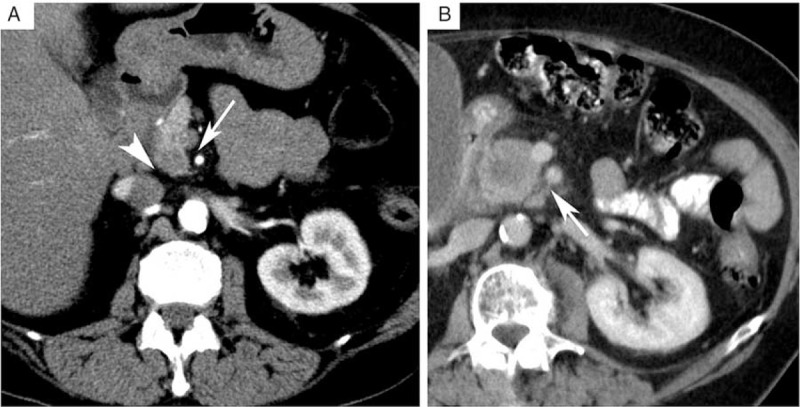
Assessment of radiologic parameters. A, Preoperative contrast enhanced multiphasic multidetector computed tomography (MDCT) demonstrating a normal *fatty tissue sheath* separating the superior mesenteric artery (SMA, arrow) and the inferior caval vein (ICV, arrowhead) from the pancreas. A small hypovascular/hypodense tumor in the pancreatic head can be seen. B, Preoperative contrast enhanced MDCT demonstrating the presence of stranding, that is increased attenuation, in the SMA fatty tissue sheath (arrow).

For preoperative staging and assessment of tumor resectability, patients routinely underwent a multiphasic multidetector computed tomography (MDCT) or a gadolinium-enhanced magnetic resonance imaging (MRI) including multiplanar sequences. Preoperative cross-sectional examinations were retrospectively reanalyzed by consensus reading by a clinically experienced radiologist and an experienced surgeon blinded for resection status. The raters evaluated each scan for 6 parameters. Thickness of the fatty tissue sheath between pancreas and superior mesenteric artery (SMA) and inferior caval vein (ICV) as well as distance between tumor and SMA / ICV was measured in millimeters (Figure 2). Furthermore, presence or absence of stranding within the fatty tissue sheaths was documented as positive or negative (Figure 2), and defined as positive when there was no fat sheath of 1 mm or more.

### Statistical Analyses

Data acquisition and statistics were carried out with MedCalc Statistical Software version 14 (MedCalc Software bvba, Ostend, Belgium). Scale parameters were expressed as median and range, ordinal and nominal variables as absolute numbers, and percent and survival data as estimates by Kaplan–Meier method. Statistical testing was performed with a 2-sided significance level of *P* = 0.05 by Kappa test for inter-rater agreement, Spearman rank test for correlation, Logrank test, and Cox proportional hazards regression for survival. Binary logistic regression and cross tabulation with calculation of sensitivity, specificity, and predictive values were used for prediction of S-status by radiologic parameters.

## RESULTS

### Patient Demographics and Operations

After clinico-pathologic reevaluation, in total n = 91 patients (41 women, 50 men) operated at a median age of 67 years (range, 36–84 years) from 2001 through 2011 for pancreatic head PDAC with sufficient tissue left for reevaluation of resection margins were included (Table 1). Most operations were pylorus-preserving pancreatoduodenectomies (87%, n = 79), and a minority were classic Whipple procedures (n = 9, 10%) and total pancreatectomies (3%, n = 3). En bloc PVR was performed in almost half of the cases (45%, n = 41).

**TABLE 1:**
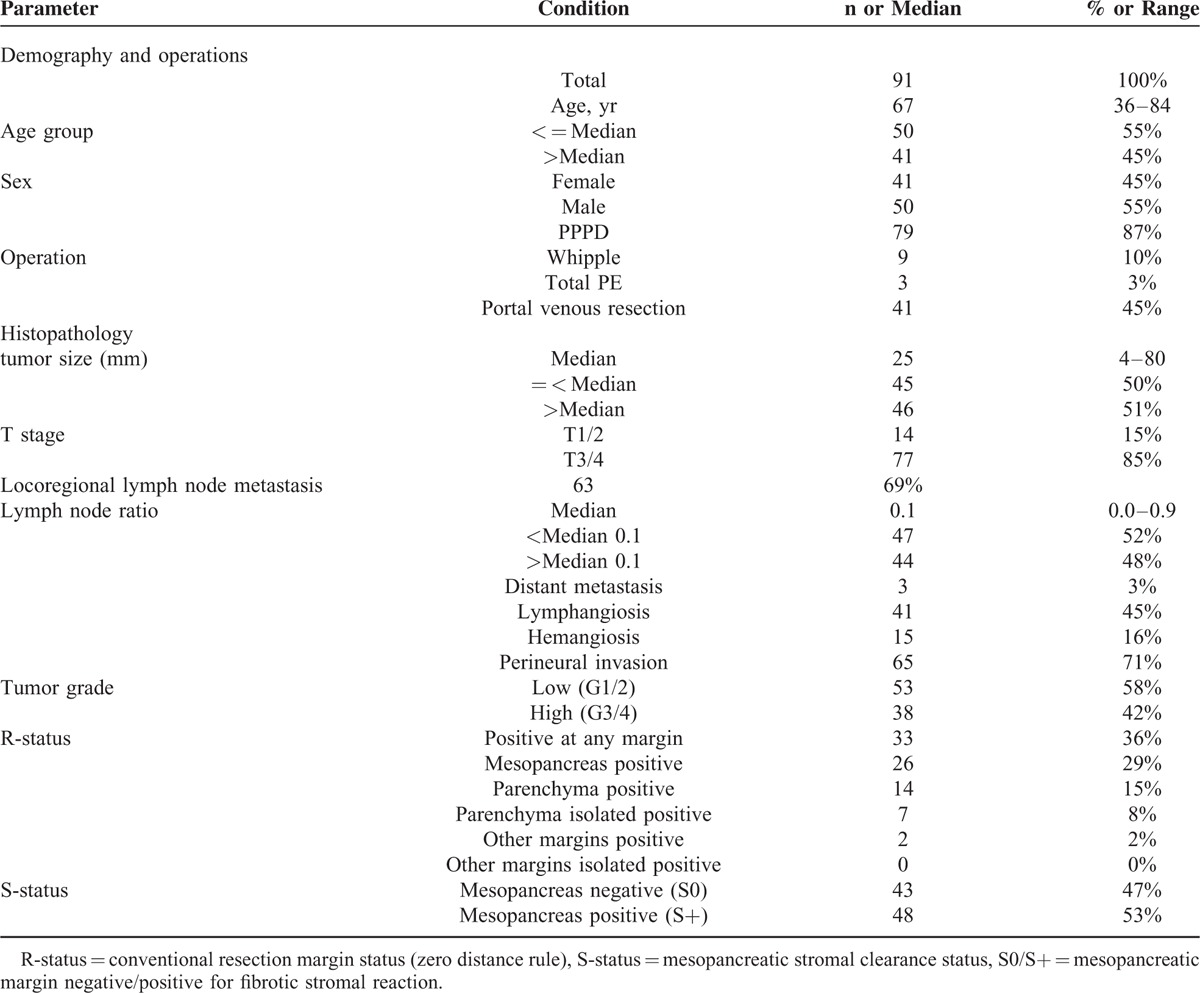
Baseline Demography, Operations and Histopathology

### Standard Histopathology

All cases were histologically confirmed as PDAC, with about half high-grade (G3/G4) tumors (Table 1). Median tumor size was 25 mm (range, 4–80 mm) and 85% (n = 77) were of T stage 3 or 4. The median number of examined lymph nodes was 14 (range, 2–43). Loco-regional lymph node metastases were detected in 69% (n = 63), with a median lymph node ratio (LNR) of 0.1 (range, 0.0–0.9). Perineural invasion (71%, n = 65) and lymphangiosis (45%, n = 41) were frequent findings, while microscopic hemangiosis (16%, n = 15) was uncommon and distant metastasis (3%, n = 3) was rare.

### Histological Margin Status

Conventionally positive margins (R+) were detected in 36 % (n = 33) of resection specimens (Table 1). Most of these were due to a positive mesopancreatic margin (79 %, n = 26). Pancreatic parenchymal margins were positive in 15% (n = 14), and other margins in only 2% (n = 2). Margin positivity did almost always involve a positive mesopancreatic margin, except for 7 cases (8% of all patients) in whom solely the pancreatic parenchymal margin was positive in definitive microscopic workup. In contrast to the conventional R-status, histological reassessment detected fibrotic stromal reaction at the mesopancreatic margins (S+) in more than half of patients (53%, n = 48). Inter-rater agreement for S-status was substantial (kappa value of 0.887, 95% confidence interval 0.791–0.983).

### Univariate Survival Analysis

Overall survival was 20 months with 48 deaths during follow-up of 91 patients (median follow-up 13 months). Among all clinico-pathologic parameters, only LNR, R-status, and S-status qualified as predictors of survival (Table 2). Lymph node ratio (cutoff 0.10) distinguished between patients with a median survival of 19 versus 29 months (*P* = 0.007, Figure 1). Current German guidelines recommend examination of at least 10 lymph nodes.^31^ However, results were unchanged when patients with less than 10 examined lymph nodes (n = 10) were excluded. The difference in survival was the longest with regard to S-status (positive vs negative, 15 vs 27 months, *P* = 0.002, Figure 2), while conventional R-status resulted in a considerably smaller difference of 20 versus 27 months (*P* = 0.03, Figure 2).

**TABLE 2:**
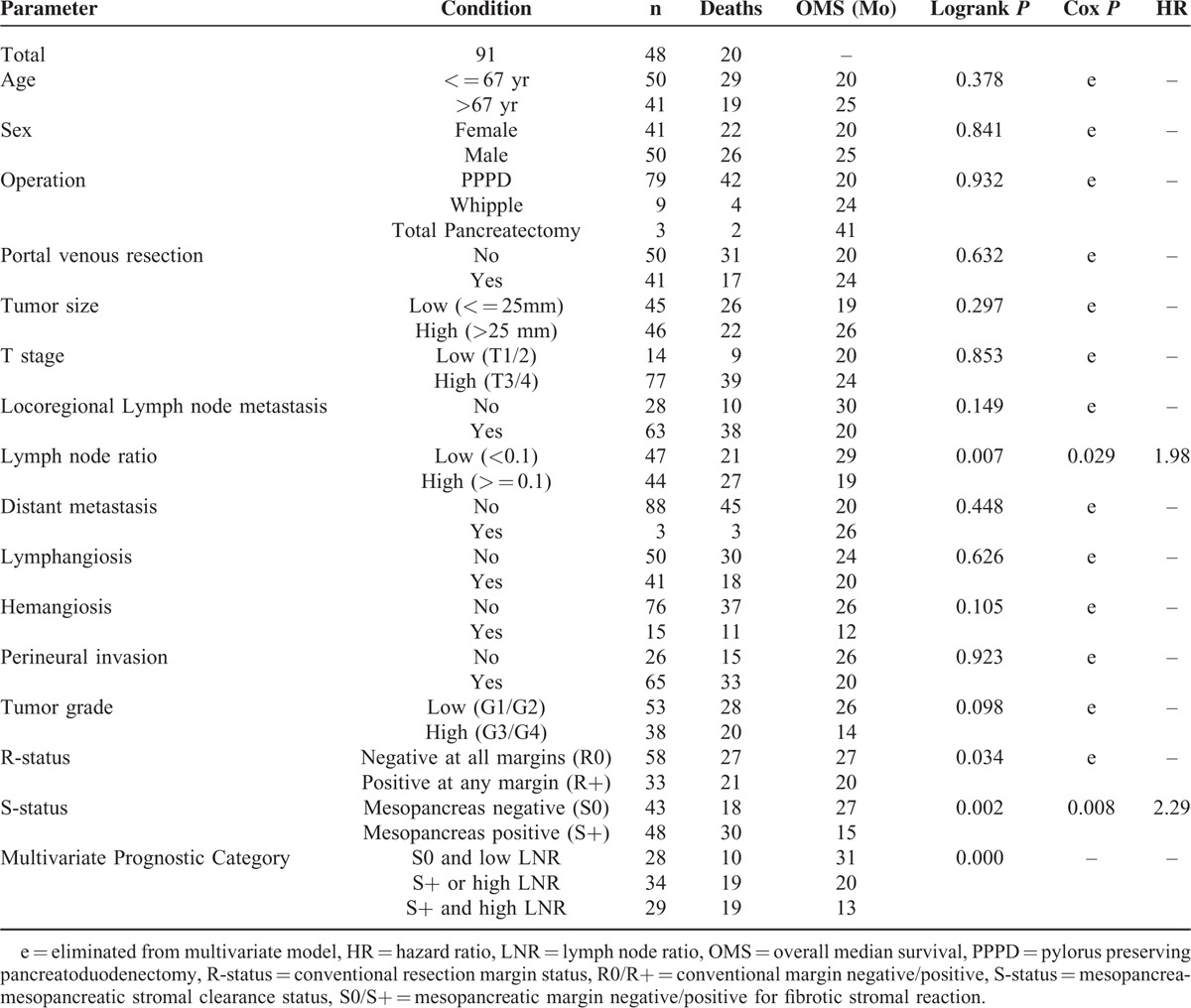
Univariate and Multivariate Survival Analysis

### Multivariate Survival Analysis

In a multivariate proportional hazards model, forward and backward elimination identified only LNR and mesopancreatic S-status as independent predictors of survival after resection of pancreatic head PDAC. Exclusion of 10 cases with less than 10 lymph nodes evaluated did not alter this result. To substantiate these results, survival was compared according to combined assessment of LNR and S-status (Table 2 and Figure 2). In patients with stromal clearance (S0) and low LNR, median survival reached 31 months, and dropped sharply when only 1 parameter (20 months) or both (13 months) were unfavorable (ie, S+ or high LNR, *P* < 0.001).

### Stromal Clearance Status in Conventionally Margin Negative Resections

**FIGURE 3:**
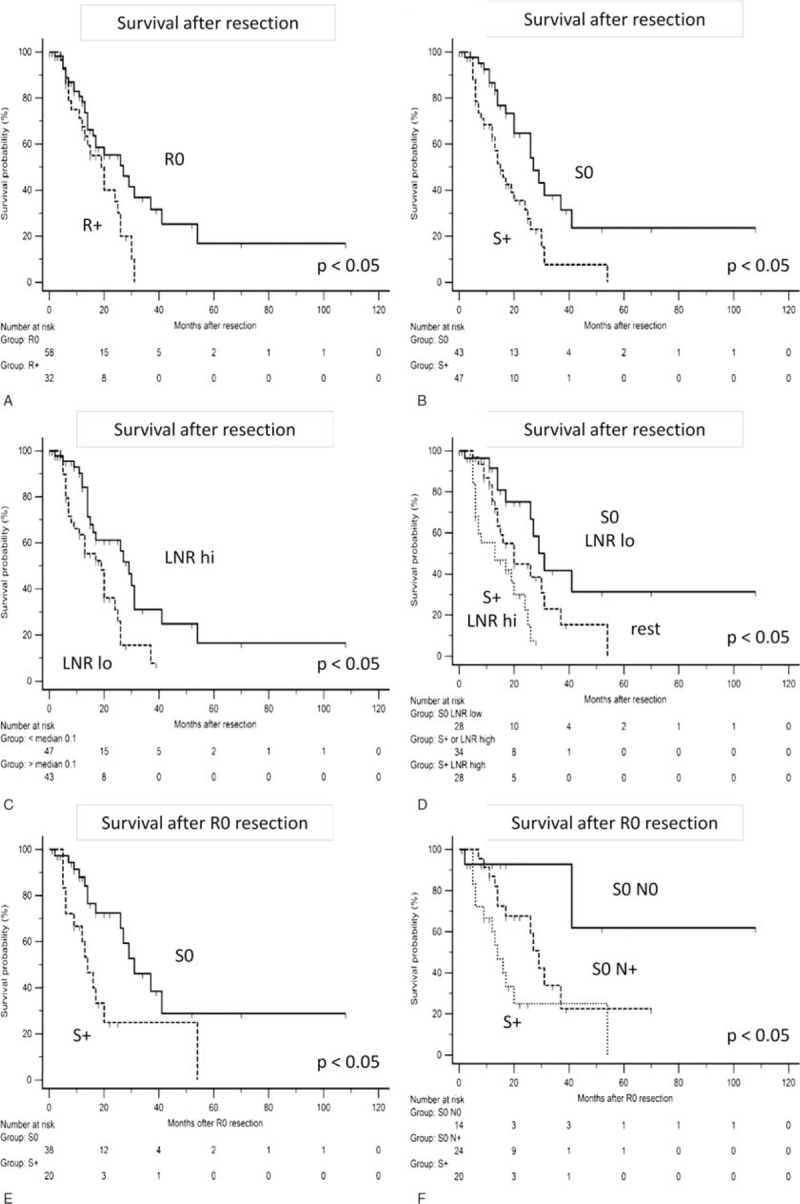
Survival analysis. A–D, Kaplan–Meier plots for comparison of survival after resection of pancreatic head cancer for (A) conventional R0 versus R+ resection, (B) mesopancreatic stroma negative (S0) versus stroma positive (S+) resection, (C) high versus low lymph node ratio (LNR), and (D) patients with S0 margins and low LNR versus patients with S+ margins and high lymph node ratio versus the rest. See also Table 2 for details. E, F, Kaplan–Meier plots for comparison of survival after R0 resection of pancreatic head cancer for (E) mesopancreatic stroma negative (S0) versus stroma positive (S+) resection and (F) patients with S0 resection and no lymph node metastasis (N0) versus S0 resection with lymph node metastasis versus S+ resection. See also Table 3 for details. LNR = lymph node ratio; N0/N+ = locoregional node metastasis absent/present; R-status = conventional resection margin status; R0/R+ = conventional margin negative/positive; S-status = mesopancreatic stromal clearance status; S0/S+ = mesopancreatic margin negative/positive for fibrotic stromal reaction, *P* values given for 2-sided Logrank test.

To further evaluate the prognostic role of the S-status, a subgroup analysis in patients with conventional margin negative resection (R0) was performed (Table 3 and Figure 3). Similarly to the results outlined above, the S-status discriminated sharply between patients with favorable and poor survival (S+ vs S0, median survival 14 vs 31 months, *P* = 0.005) in this smaller (n = 58) subgroup with a median overall survival of 27 months. Locoregional lymph node metastasis (N0/N+) further subdivided the prognostic categories: Survival after S0 resection with positive locoregional lymph nodes was 29 months, while median survival was not even reached during follow-up in the small group of patients with S0 resection and negative lymph nodes (n = 14, *P* = 0.01) (Table 3 and Figure 3).

**TABLE 3:**
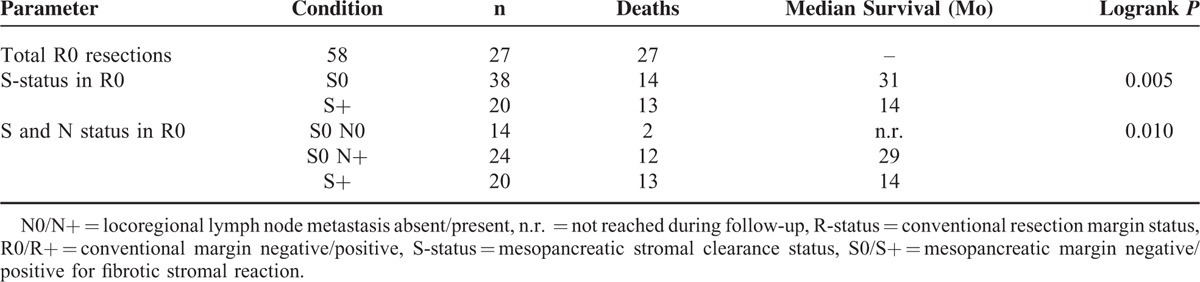
Survival Analysis in the R0 Subgroup

### Correlation of Mesopancreatic Stromal Clearance With Tumor Biology

To assess the biologic role of the S-status, correlation analysis between S-status and other histopathological parameters (supplemental Table S1) was carried out. Only conventional R-status and LNR showed significant positive correlation with S+ resection. There was no apparent correlation with tumor size or markers of aggressive disease like lymphangiosis, hemangiosis, or perineural invasion.

### Correlation of Stromal Clearance Status With CRM Concept

The closest distance between tumor cells and resection margin was measured at the mesopancreatic margin and categorized according to the CRM concept ^17^ as positive (CRM+) when tumor cells were found within 1 mm from the resection margin. There was a strong correlation between the categories S+ and CRM+, with 81% of the S+ cases also being CRM positive and 91% of the S0 cases being CRM negative (Table 4, *P* < 0.001 for 2-sided Spearman Rank correlation).

**TABLE 4:**
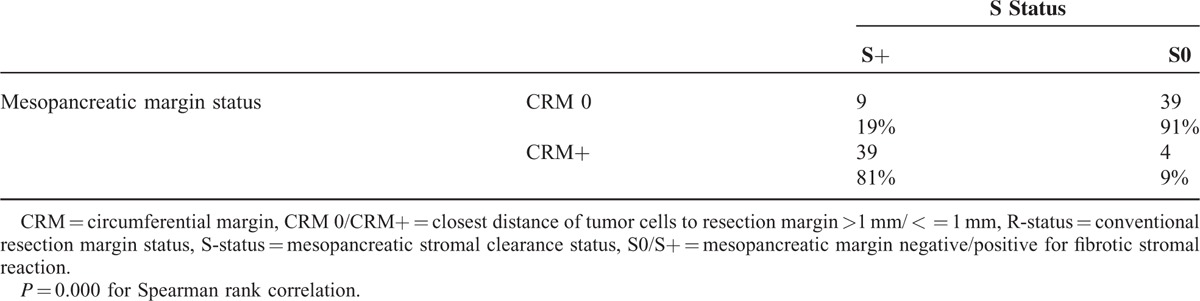
Correlation Between S Status and CRM Concept

### Prediction of Stromal Clearance Status by Radiographic Parameters

As fibrotic changes in the mesopancreatic fatty tissue can be visualized by stranding in MDCT or MRI, we evaluated a multivariate model for the prediction of mesopancreatic S-status (supplemental Table S2). Nineteen patients had to be excluded from this analysis due to unavailable preoperative imaging sets or insufficient image quality.

In univariate logistic regression analysis, thickness and stranding of the SMA fat sheath were significant predictors of S-status, while stranding of the fat plane between ICV and pancreas reached a statistical trend (*P* = 0.05). In a multivariate logistic regression model including these 3 parameters, only SMA fat sheath stranding qualified as independent predictor. Cross tabulation analysis disclosed positive and negative predictive values of 77% and 66%, with an overall accuracy of 71% for prediction of S-status by SMA fat sheath stranding (supplemental Table S2).

## DISCUSSION

Prognosis of PDAC remains poor even in patients with radical surgical resection, due to local and systemic recurrence.^2,3,32^ Several hypotheses are usually given to explain these clinical observations. On the one hand, PDAC is supposed to have an intrinsic aggressive biology featuring highly invasive cancer cells,^1^ discontinuous growth,^33^ perineural spread,^34^ as well as high metastatic potency.^8^ Nevertheless, data to support the a priori assumption that PDAC is intrinsically more aggressive than other carcinomas is very scarce at best.

On the other hand, radical wide surgical resection is anatomically impossible and therefore successful surgical resection has been conventionally defined as the achievement of histopathologically tumor cell free margins (R0 resection).^17^ Positive resection margins in pancreatoduodenectomy specimen are most frequently found in the retroperitoneal tissue dorsal to the pancreatic head and neck and toward the superior mesenteric artery.^14–17^ This area has previously been coined “mesopancreas.”^10,11^ Other authors refer to it as the retroperitoneal, medial, posterior, uncinate, or superior mesenteric artery margin.^14–17^ We prefer to use the term mesopancreas because it describes well its development and function.^10^ In our series this margin was routinely marked by the surgeon and was found to be the most critical margin in terms of conventional margin status.

Given the anatomic complexity of pancreatic head resection, it is not surprising that margin status derived from nonstandardized histopathologic workup protocols frequently failed to achieve prognostic value.^17^ Detection of tumor positive margins according to the few currently standardized protocols essentially relies on 2 measures: extensive specimen workup by serial tissue slicing with resection plain inking and definition of a negative margin by a minimum distance of tumor cells from the inked resection plain. These concepts were developed in analogy to the circumferential margin workup of rectal cancer resection specimens.^17^ Using these novel protocols, over 80% of pancreatoduodenectomy specimen were found to yield positive margins,^17^ providing a simple yet important explanation for the notorious failure of surgical therapy.

It remains unclear whether the more extensive circumferential workup by serial tissue slicing or the assessment of the distance between tumor cells and resection margin contributes to this recently increased detection of margin positive resection. The definition of a margin negative resection remains a matter of debate. As a biological rationale for a distance cut-off, the dispersed growth of PDAC has been suggested.^17,33^ In the absence of data defining a widely accepted cut-off, most authors rely on a minimum tumor cell to margin cut-off of 1 mm, in analogy to the CRM concept in rectal cancer.^17^

The current study evaluates a clinically and biologically inspired classification of margin assessment in PDAC. Clinical experience shows that a strong fibrotic stromal reaction can often be observed in the peritumoral mesopancreatic tissue, sometimes even necessitating sharp dissection, especially from major blood vessels. Given these clinicopathologic experiences and the apparent prominent role of the desmoplastic stroma in PDAC, we tested the simple mechanistic hypothesis that clearance of the peritumoral fibrotic stroma determines the oncologic outcome of resection.

As a potential drawback, our study was performed retrospectively on the basis of a patient cohort that had not been assessed by 1 of the novel extensive standard workup protocols. Nevertheless, the conventional margin status (R-status) achieved statistically significant influence on overall survival after resection. These results highlight that a focused standard approach, concentrating on cooperation of surgeons and pathologists, results in clinically valid margin assessment. It further confirms the results of other authors ^14,35–38^ demonstrating the mesopancreatic margin as the clinically most influential of all margins in pancreatoduodenectomy. Only less than 10% of resections were R+ because of isolated involvement of the pancreatic parenchymal margin, and only 2% of other margins were even found to be positive.

While roughly one-third of resections were margin positive (R+) by conventional means, more than half of all cases were retrospectively found to have stromal positive (S+) mesopancreatic resection margins in re-evaluation. Furthermore, only stromal clearance at the mesopancreatic margin (S0 resection) had a very significant and strong positive influence on overall survival after resection. Of note, lymph node ratio was the other independent prognostic parameter in multivariate analysis. S-status additionally provided a clear prognostic subcategorization of the patient group with conventional R0 status. The small subgroup of patients with lymph node negative disease and mesopancreatic stromal clearance displayed a very favorable prognosis rarely observed in PDAC.

The positive correlation of S+ resection with lymph node ratio may suggest that S-status is also related to intrinsic tumor aggressiveness. It might be speculated that more aggressive tumors display more effective lymphatic dissemination on the one hand and more diffuse growth on the other hand, rendering them less amenable to S0 resection. Similar observations have been reported by other authors for the correlation of R-status with lymphangiosis and lymph node metastasis.^39,40^ Further biologic interpretation remains to be examined. On the basis of our data we cannot decide whether peritumoral fibrotic stroma left in place results in cancer recurrence or whether stromal clearance is just a sensitive surrogate marker of undetected dispersed cancer cells left behind.

Some authors have recently suggested total mesopancreatic resection,^41–43^ which involves paraaortic lymphadenectomy of stations 16a and 16b as well as circular lymphadenectomy around the superior mesenteric artery.^11^ However, in the absence of evidence for a survival benefit, but instead increased morbidity with extended lymphadenectomy in randomized trials,^44^ total mesopancreatic resection is not advocated in the current guidelines of the International Study group for Pancreatic Surgery (ISGPS).^44^ At our institution, only standard lymphadenectomy was performed during pancreatoduodenectomy, corresponding to the current ISGPS guidelines. This includes en bloc resection of mesopancreatic tissue dorsal to the pancreatic head and neck and to the right side of the superior mesenteric artery.^44^

Theoretically, in case of macroscopic tumor infiltration, total mesopancreatic resection could result in stroma-negative margins and survival benefit. This hypothesis would have to be tested in a randomized trial. Currently, however, taking into account the data from randomized controlled trials on extended lymphadenectomy,^44^ we do not feel that our findings advocate routine extended lymphadenectomy/total mesopancreatic resection.

We further demonstrate feasibility of correct preoperative prediction of stromal clearance on the basis of standard cross-sectional imaging in over 70% of patients. This issue has not been assessed for the other novel workup protocols ^17,23^ and should be validated, ideally in a prospective randomized fashion. According to our data, about half of all patients selected for upfront pancreatic head cancer surgery can be expected to have no stromal clearance from resection. Consequently, measures to down-stage the tumor before resection may be advocated. In current clinical practice, only so-called borderline resectable PDAC is a widely accepted indication for neoadjuvant treatment. However, our radiologic criteria are more stringent than those currently proposed to define borderline resectable PDAC.^18,22^ In summary, our data may suggest that a redefinition of borderline resectable disease and broader application of neoadjuvant treatment may be necessary to meet the clinical challenge of pancreatic cancer, when the above-mentioned criteria are present. The proposed mesopancreatic stromal clearance status proved to be a very powerful and clinically valid prognostic factor in patients receiving resection of pancreatic head cancer.

## Supplementary Material

Supplemental Digital Content
