# Supplementary material for: Mesopancreatic Stromal Clearance Defines Curative Resection of Pancreatic Head Cancer and Can Be Predicted Preoperatively by Radiologic Parameters: A Retrospective Study
Source: Medicine (Baltimore). 2016 Jan 22;95(3):e2529. doi: 10.1097/MD.0000000000002529 (PMC4998270; doi:10.1097/MD.0000000000002529)
Supplement: Supplemental Digital Content [file medi-95-e2529-s001.docx]

**Table S1. S status correlation analysis.**

Abbreviations: S status mesopancreatic stromal clearance status, S0/S+ mesopancreatic margin negative/positive for fibrotic stromal reaction, R status conventional resection margin status, CC correlation coefficient, p value given for Spearman rank correlation.

| correlation with positive S status (S+) | | |
| --- | --- | --- |
| parameter | CC | p |
| R status positive | 0.485 | <.001 |
| tumor size | 0.076 | .47 |
| T stage | -0.099 | .35 |
| locoregional lymph node metastasis | 0.084 | .43 |
| lymph node ratio | 0.255 | .02 |
| distant metastasis | 0.051 | .63 |
| lymphangiosis | 0.105 | .32 |
| hemangiosis | 0.124 | .24 |
| perineural invasion | -0.014 | .90 |
| tumor grade | 0.132 | .21 |

**Table S6. Preoperative prediction of S status by radiologic parameters**

uni/multivariate p value derived from binary logistic regression; Abbreviations: ICV inferior caval vein, SMA superior mesenteric artery, TU tumor, S status mesopancreatic stromal clearance status, S0/S+ mesopancreatic margin negative/positive for fibrotic stromal reaction, CI confidence interval, e excluded from analysis, fat [mm] thickness of fat sheath separating pancreas and blood vessel, TU distance [mm] distance of blood vessel wall from tumor

| **logistic regression analysis** | | | | |
| --- | --- | --- | --- | --- |
| parameter | OR | univariate p | | multivariate p |
| VCI fat [mm] | 1.1 | .51 | | e |
| VCI fat stranding | 2.7 | .05 | | .16 |
| VCI TU distance [mm] | 1.0 | >.99 | | e |
| SMA fat [mm] | 0.8 | .01 | | .57 |
| SMA fat stranding | 6.6 | .001 | | .01 |
| SMA TU distance [mm] | 0.9 | .11 | | e |
| **prediction of S+ status by SMA fat sheath stranding** | | | | |
| actual S status | predicted S status | | correct prediction | |
|  | 0 | 1 |  |  |
| S0 | 27 | 7 | 79% (specificity) | |
| S+ | 14 | 24 | 63% (sensitivity) | |
| positive predictive value | | 77% | | |
| negative predictive value | | 66% | | |
| cases correctly predicted overall | | 71% | | |
